# DNA Methylation Inhibits the Expression of CFSH in Mud Crab

**DOI:** 10.3389/fendo.2020.00163

**Published:** 2020-04-09

**Authors:** Qingling Jiang, Dongdong Lin, Huiyang Huang, Guizhong Wang, Haihui Ye

**Affiliations:** College of Ocean and Earth Sciences, Xiamen University, Xiamen, China

**Keywords:** crustacean female sex hormone, promoter, DNA methylation, epigenetics, mud crab

## Abstract

Crustacean female sex hormone (CFSH) is a key regulator of crustacean sex differentiation. The expression of *Sp-CFSH* in the mud crab *Scylla paramamosain* showed a tissue-specific and gender-variant pattern. To explore the role of DNA methylation in *Sp-CFSH* expression, the 5′-flanking region of *Sp-CFSH* was cloned, and one CpG island containing 12 CpG sites was found. Results of sodium bisulfite sequencing and methylated DNA immunoprecipitation showed that CpG island methylation was stable in the eyestalk ganglion during ovarian development of the females, which was significantly lower than that in the muscle of the females and in the eyestalk ganglion of the males. Such results suggested that the involvement of DNA methylation in regulating *Sp-CFSH* expression followed an eyestalk ganglion-specific and gender-variant pattern. The analysis of CpG dinucleotide site methylation and activity of the site-directed mutation (SDM) reporter vector further demonstrated that methylation inhibited *Sp-CFSH* expression by blocking the binding of transcription factor Sp1. The finding suggested for the first time the involvement of CpG methylation in the regulation of *Sp-CFSH* expression.

## Introduction

The regulation of eukaryotic gene expression is complex and rigorous, and influenced by various levels, such as the genetic level of DNA regulation, epigenetic level of chromatin regulation, the post-transcription level of RNA regulation, the translation and protein processing regulation, phosphorylation and acetylation of protein, heat shock protein regulation, etc. (1–4). For a given gene, intergenic regions play an important role in the regulation (5–7). The eukaryotic promoter, a region with various regulatory elements, is located upstream of the gene and determines the level of expression via different regulatory factors (8, 9).

Methylation of genomic DNA, as a major epigenetic modification, generally modulates transcription by influencing the binding of regulatory factors to regulatory elements (10). In vertebrates, DNA methylation is throughout the genome and involved in silencing of gene expression during cellular differentiation and development (11–15). CpG island is the major target for methylation and plays a vital role in inhibiting gene expression (16). Dynamic methylation pattern of CpG island in a core promoter is involved in the regulation of gene expression (17). In insects, DNA methylation is restricted to the transcribed regions and primarily involved in behavioral plasticity and social behavior (17–22). Unlike mammals and insects, studies on DNA methylation are relatively rare in crustaceans and have so far been reported only in the water flea, *Daphnia magna* and *Daphnia pulex*. In *D. magna*, DNA methylation levels can be affected by Zn exposure and entailed at different histories (23, 24). In *D. pulex*, the 5-methyl-cytosine (5-mC) and 5-hydroxymethyl-cytosine (5-hmC) can inhibit the expression of the cullin-associated NEDD8-dissociated 2 (*Cand2*) gene, cytochrome C oxidase subunit IV (*Cox4*) gene, and juvenile hormone epoxide hydrolase 1 (*Ephx1*) gene (25).

Crustacean female sex hormone (CFSH), a key regulator of sex differentiation, has been shown to regulate reproductive processes such as the development of sexually dimorphic traits and expression of androgenic gland hormone (IAG) (26–28). The ontogenic of *CFSH* expression was detected in embryos at hatching stage in the blue crab, *Callinectes sapidus* (26). In the mud crab, *Scylla paramamosain, Sp-CFSH* expression shows tissue-specific and gender-variant pattern, which was exclusively expressed in the eyestalk ganglion and higher in mature females than in males (27). In addition, *Sp-CFSH* expression was dynamic during the development of the androgenic gland (AG) in *S. paramamosain*, which was maintained at high levels at the early stage (stages I and II) and then reduced significantly at the mature stage (stage III) (27).

To explore the regulatory mechanism of expression, the 5′-flanking region of *Sp-CFSH* was cloned and analyzed. Sodium bisulfite sequencing and methylated DNA immunoprecipitation were used to investigate the involvement of DNA methylation in the regulation of *Sp-CFSH* expression. Moreover, CpG dinucleotide site methylation and activity of the site-directed mutation (SDM) reporter vector were analyzed to demonstrate the regulatory mechanism that methylation inhibits *Sp-CFSH* expression.

## Materials and Methods

### Animal Sources

Mud crabs (*S. paramamosain*) were obtained in March from a local market in Xiamen, Fujian Province, China. They were reared in tanks (temperature: 27 ± 2°C; salinity: 26 ± 1 ppm) for a week and fed with the meat of the white Pacific shrimp, *Litopenaeus vannamei*. The female crabs (carapace width 4.9–12.5 cm, body weight 115–382 g) and male crabs at stage III of AG development (*n* = 3, carapace width 9.3–11.8 cm, body weight 230–372 g) (27, 29) were anesthetized; the muscle and eyestalk ganglion were dissected to prepare the genomic DNA. In addition, the eyestalk ganglion of female crabs (*n* = 8) were collected to analyze the *Sp-CFSH* expression. Ovarian development was distinguished according to the morphological characteristics and confirmed by histological observation (30, 31). All the animals used in this study have been approved by the Animal Ethics Committee of Xiamen University.

### Cloning of the 5′-Flanking Sequence of *Sp-CFSH*

The genomic DNA was purified from muscle using the Universal Genomic DNA Extraction kit (TaKaRa, Japan). Tail-PCR was employed to clone the 5′-flanking region of *Sp-CFSH* according to the manufacturer's instructions (32, 33). The gene-specific primers (SP1-SP3 and SP4-SP6) (Table 1) were designed based on the sequence of *Sp-CFSH* (27) and used to clone the 5′-flanking region with random primers (AP1-4) in the Genome Walking kit (TaKaRa, Dalian, China).

**Table 1:** The primers used in the present study.

**Name**	**Sequence (5^**′**^ → 3^**′**^)**	**Application**
SP1	CATGTGTCCTATGATGGAGGAACG	Tail-PCR
SP2	GCAAGAAATGCTGGACACGTGAAG	
SP3	AGGGAAGTTCTGTTCTGCTTCAT	
SP4	GTAGTAAATCCCAGGTGCGTAAAG	
SP5	GACAACCTACTCAGTAACATCG	
SP6	GCGAGCGACAAGGCACAGTAAT	
*Sp-cfsh*-QF	cggGGTACCAATCGGCATTTAGGTTTATTTGGTC	Cloning the 5**′**-flanking sequence
*Sp-cfsh*-QR	cgcGGATCCTGTAAGCCTTAGGGAAGTTCTGTT	
*Sp-cfsh*-F	CGTGTCCAGCATTTCTTGCAGTACC	qRT-PCR
*Sp-cfsh*-R	TCATGTGTCCTATGATGGAGGAACG	
*Sp-ak*-F	TTCCTCCACCCTGTCCAACC	
*Sp-ak*-R	GAAGCGGTCACCCTCCTTGA	
BSP-F	TTTTTAATTAAAATAAATATTTGATT	Determination of DNA methylation
BSP-R	ATAAAAATTTTAACATCCATCTCAC	
p*sp-cfsh*-F	cggGGTACCTATTTACATGAAGATGCAATGGGCT	SDM
p*sp-cfsh*-R	cccAAGCTTCCTTAGGGAAGTTCTGTTCTGCTTC	
m*sp-cfsh*-F	ACAAACACCTGACTCTACCGCGCTGGTT	
m*sp-cfsh*-R	AACCAGCGCGGTAGAGTCAGGTGTTTGT	

### Bioinformatics Analysis

NNPP (http://www.fruitfly.org/seq_tools/promoter.html) was used to predict the core promoter region and transcription initiation site with the minimum promoter score of 0.75.

AliBaba 2.1 (http://gene-regulation.com/pub/programs/alibaba2/index.html) with defaulted parameters was applied to predict the potential transcription factor binding sites.

MethPrimer (http://www.urogene.org/cgi-bin/methprimer/methprimer.cgi) was applied to predict the CpG island, which is defined using the following criteria (34): island size with minimum length 200 bp, GC content <55%, and ratio CpG observed/expected < 0.60.

### *Sp-CFSH* Promoter Activity Analysis

The 5′-flanking sequence of *Sp-CFSH* was sequenced and cloned into pMD19-T vector (TaKaRa, Dalian, China). After digesting with KpnI and HindIII (TaKaRa, Dalian, China), the 5′-flanking sequence of *Sp-CFSH* was ligated to a promoterless enhanced green fluorescent protein (EGFP) report vector (pEGFP-1; YouBio, Changsha, China). The recombinant vector (pEGFP-pCFSH) was used to test promoter activity by transfecting HEK293FT cells. The HEK293FT cells were obtained from the China Center for Type Culture Collection, Wuhan. They were cultured in high-glucose DMEM (HyClone, USA) with 10% FBS (Gibco, USA), 1% 100 penicillin–streptomycin (Gibco, USA) and incubated at 37°C under 5% CO_2_. Cells were plated on 24-well plates overnight, and then transient transfections were performed using Lipofectamine^TM^ 2000 Reagent (Invitrogen, China) with 1 μg of pEGFP-pCFSH following the manufacturer's protocols. pEGFP-1 and pEGFP-N1 were used as the negative control and mock transfection, respectively. pEGFP-N1 was gifted by Dr. Kejian Wang of College of Ocean and Earth Sciences, Xiamen University, China.

### *Sp-CFSH* Expression Analysis

Total RNA was extracted from eyestalks using TRIzol Reagent (Invitrogen, USA), and the quality was detected using NanoDrop-2000 spectrophotometer (Thermo Fisher Scientific) according to the manufacturer's instructions. The cDNAs were synthesized using 1 μg of total RNA and TransScriptII One-step gDNA Removal and cDNA Synthesis SuperMix kit (TransGen, Beijing, China) according to the manufacturer's protocol. The cDNAs were diluted four times for qRT-PCR analysis. The qRT-PCR was performed with 7500 Fast Real-Time PCR (Applied Biosystems), with the reaction volumes of 10 μl of 2 × PCR Master Mix with SYBR Green, 2 μl of dilute cDNA, 0.8 μl of each primer (1 mM), 6.4 μl of water; the reaction was performed under the following conditions: 94°C for 10 min followed by 40 cycles of 94°C for 30 s, 58°C for 30 s, 72°C for 30 s, and a final extension at 72°C for 10 min. *Arginine kinase* (*AK*; GenBank accession number: JQ031765) was used as reference, and the primers used for qRT-PCR are listed in Table 1.

### Determination of DNA Methylation Level Using Sodium Bisulfite Genomic Sequencing

The bisulfite modification of genomic DNA was performed using ZYMO EZ DNA Methylation-Gold kit (D5005, Zymo Research, USA) according to the manufacturer's instructions. PCR was carried out with the bisulfate-specific primers (Table 1), and products were purified and cloned into the pMD19-T vector. After transfection, 10 positive clones were selected and sequenced. The analysis was performed as previously described (17).

The rate of promoter methylation was calculated by the formula I^Me^/10, where I^Me^ and 10 represent the number of the methylated promoters and sequenced promoters, respectively.

Average methylation of promoter was calculated by the formula $\sum_{i=1}^{N}\text{Si/}12\text{/N}$, where S_i_, 12, and N represent the number of the methylated dinucleotides site, 12 sites of CpG island, and methylated promoters, respectively.

Average methylation levels of the CpG dinucleotide site was figured by the formula S^Me^/10, where S^Me^ and 10 represent the number of methylated dinucleotides site and 10 dinucleotides site of sequenced promoters, respectively.

The results from at least three independent experiments were quantified and averaged.

### Methylated DNA Immunoprecipitation (MeDIP)

MeDIP analysis was performed as previously described with minor modifications (35). The genomic DNA was sonicated (15 min on ice with 15 s on/off intervals; Branson Sonifier S-450D, USA) to yield DNA fragments from 200 to 500 bp in length. One microgram of fragmented DNA was heated and denatured to produce a single-stranded DNA, then immunoprecipitated with l μg of anti-5mC antibody (ab10805; Abcam, UK) or with 1 μg of normal mouse IgG (as negative control) at 4°C for 12 h. Pre-cleared Protein A/G PLUS-Agarose (sc-2003; Santa Cruz) immunoprecipitated antibody/DNA complexes were washed away unless specifically bound, and finally resuspended with 500 μl of digestive buffer containing proteinase K. The DNA fragments was purified with phenol chloroform extracting and ethanol precipitated, then resuspended in 50 μl of Tris buffer (10 mM Tris, pH 8.5). Finally, 2 μl of DNA fragments was used for analyzing the methylated rate of the 5′-flanking region by PCR.

### Promoter Activity Analysis of the Binding Site of Sp1 With Site-Directed Mutagenesis (SDM)

SDM was achieved by overlap extension PCR reactions with primers containing the mutational bases and was used to identify the function of transcription elements. p*sp-cfsh*-F/m*sp-cfsh*-R and p*sp-cfsh*-R/m*sp-cfsh*-F (Table 1) were used to amplify p*sp-cfsh*-1 and p*sp-cfsh*-2 with procedure as follows: 94°C for 5 min; 34 cycles of 94°C for 30 s, 57°C for 30 s, 72°C for 1 min, followed by the final extension at 72°C for 10 min using the ABI 2720 Thermal Cycler (Applied Biosystems, USA). The second reaction was carried out with amplification system that contained 2.5 μl of 10 × Ex Taq Buffer (TaKaRa, Dalian, China), 2 μl of dNTP, 0.25 μl of Ex Taq, 1 μl of p*sp-cfsh*-1, 1 μl of p*sp-cfsh*-2, and 16.65 μl of water, under the conditions 94°C for 5 min; 10 cycles of 94°C for 40 s, 57°C for 1 min, 72°C for 1 min, followed by 20°C for 5 min; Then, 0.8 μl of p*sp-cfsh*-F and 0.8 μl of p*sp-cfsh*-R were added to the amplification system and reaction under the conditions 35 cycles of 94°C for 40 s, 57°C for 1 min, 72°C for 1 min, followed by 72°C for 10 min. The products were purified and digested with KpnI and HindIII (TaKaRa, Dalian, China), then inserted into pGL3-Basic vector. The reporter vectors were transiently transfected into HEK293FT cells, and the relative luciferase activity was evaluated by the Dual-Luciferase Reporter Assay System (Promage, USA). pRL-TK vector was co-transfected to normalize the transfection efficiency. The reporter vector with normal transcription element and pGL3-basic were employed as the control and the negative control, respectively.

### Statistical Analysis

The data are presented as means ± standard error of mean (SEM) of three or six independent experiments. The statistical evaluation was performed in the GraphPad Prism 6 software package (San Diego, CA, USA). Statistical analysis among groups was conducted with one-way ANOVA test, and a value of *p* < 0.05 was considered statistically significant.

## Results

### Quantitative Analysis of *Sp-CFSH* Expression

**Figure 1:**
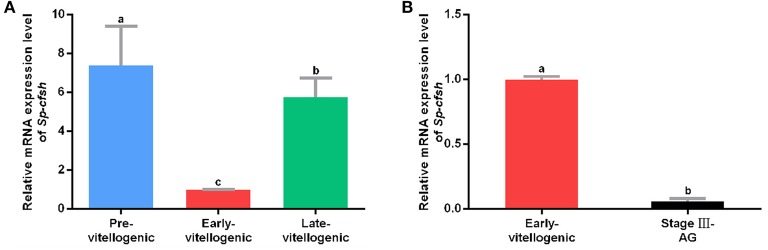
Quantitative analysis of *Sp-CFSH* expression. **(A)** Expression of *Sp-CFSH* at different stages of ovarian development. **(B)** Expression of *Sp-CFSH* in two sexes. The eyestalk ganglion of females at pre-vitellogenic stage, early-vitellogenic stage, and late-vitellogenic stage are designated as Pre-vitellogenic, Early-vitellogenic, and Late-vitellogenic, respectively, and the eyestalk ganglion of males at stage III of AG development is designated as Stage III-AG. The data are presented as mean ± SEM (*n* = 6) with different letters indicating statistical significance at *p* < 0.05.

A previous study showed that the expression of *Sp-CFSH* was dynamic during the development of AG in males. It was high at the early stage (stages I and II) and significantly decreased at the mature stage (stage III). To examine the expression of *Sp-CFSH* during ovarian development, cDNA was derived and analyzed from the eyestalk ganglion at the pre-vitellogenic stage (Figure S1A), early-vitellogenic stage (Figure S1B) and late-vitellogenic stage (Figure S1C). The results showed that the expression of *Sp-CFSH* was significantly high at the pre-vitellogenic stage and late-vitellogenic stage compared with that of the early-vitellogenic stage (Figure 1A). Females at the early-vitellogenic stage and males at the mature stage were chosen to compare the expression of *Sp-CFSH* in the two sexes, and the results showed that the expression of *Sp-CFSH* in females was significantly higher than that in males (Figure 1B).

### 5′-Flanking Sequence of *Sp-CFSH*

**Figure 2:**
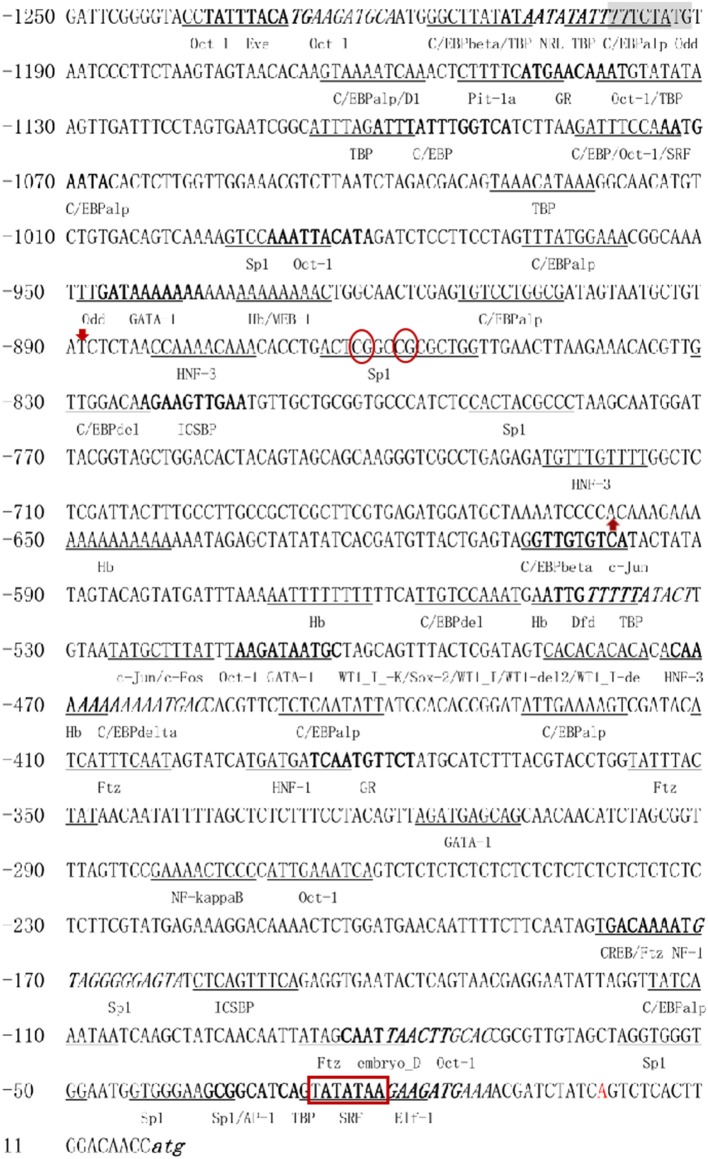
The 5′-flanking region and putative *cis*-acting elements. The deduced transcriptional start site (**A**) is marked by red and defined as position 1. The nucleotide sequence is numbered on the left. The TATA box is marked in bold with red border. The putative transcription factor binding sites are marked by underlined, bold, or italic. Red arrows mark the beginning and end of CpG island, CpG-1 and CpG-2 are marked in red oval.

**Figure 3:**
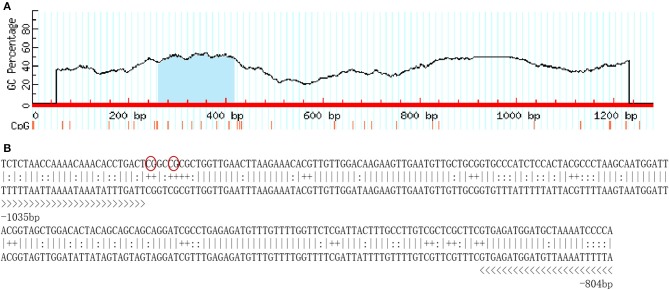
The CpG island in the 5′-flanking sequence of *Sp-CFSH*. **(A)** CpG island predicted by MethPrimer (Island size > 100, GC Percent > 50.0, Obs/Exp > 0.6). **(B)** The sequence of CpG island. Twelve CpG dinucleotides are marked with “+,” and primers used for determination of DNA methylation are marked with “>.” CpG-1 and CpG-2 are marked in red oval.

A total of 1,250-bp 5′-flanking regions (GenBank accession number: MN938502) were obtained from the transcriptional start site (TSS). The core promoter was located between −40 bp and +10 bp and contained the TSS and a TATA box (21 bp upstream of the TSS). Forecast analysis identified some transcription factor binding sites, such as Sp1, GATA-1, Wt1, Sox-2, C/EBPalp, and c-Jun (Figure 2). One CpG island was found at −687 to −918 bp and contained 12 CpG sites (Figure 3), in which CpG-1 and CpG-2 were located in the binding site of the transcription factor Sp1.

### *Sp-CFSH* Promoter Activity

**Figure 4:**
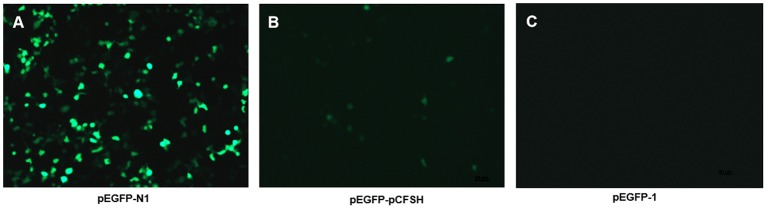
The expression of green fluorescence (EGFP) in HEK293FT cells. EGFP was detected in HEK293FT cells transfected with pEGFP-N1 **(A)**, pEGFP-pCFSH **(B)**, and pEGFP-1 **(C)**. pEGFP-N1 and pEGFP-1 were used as positive control and negative control, respectively.

The pEGFP-N1 contains the promoter of cytomegalovirus (CMV), as a positive control, which can effectively turn on the expression of EGFP and show a strong fluorescence (Figure 4A). Figure 4B showed the HEK293FT cells transfected with pEGFP-pCFSH, and the green cells demonstrated the promoter activity of the 5′-flanking sequence. The HEK293FT cells transfected with pEGFP-1 served as the negative control, and no fluorescence was detected (Figure 4C).

### CpG Island Methylation

**Figure 5:**
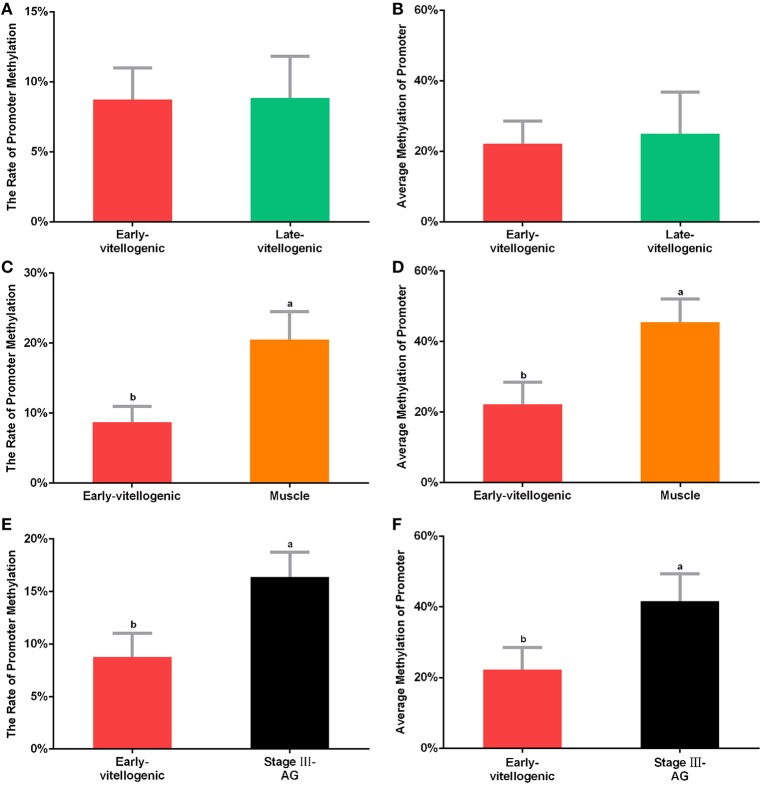
Methylation of CpG island. The percentage of methylated CpG island in the eyestalk ganglion of females at early-vitellogenic stage compared with the eyestalk ganglion at late-vitellogenic stage **(A)**, the muscle of females **(C)**, and the eyestalk ganglion of males **(E)**. The average methylation of CpG island between the eyestalk ganglion of females at early-vitellogenic stage and the eyestalk ganglion at late-vitellogenic stage **(B)**, the muscle of females **(D)**, the eyestalk ganglion of males **(F)**. Different letters represent significant difference between groups (*p* < 0.05, one-way ANOVA followed by the Tukey's *post hoc* test). Each bar represents the mean ± SEM (*n* = 3).

CpG island is known as the main target of methylation. In order to detect whether methylation is involved in the regulation of *Sp-CFSH* expression, sodium bisulfite sequencing was used to analyze the CpG island. The results showed that the level of CpG island methylation was similar in females at the stage between early-vitellogenic and late-vitellogenic, suggesting that CpG island methylation may be not involved in the regulation of *Sp-CFSH* expression during ovarian development. Compared with the eyestalk ganglion in females, CpG island methylation was significantly higher in the muscle of females and the eyestalk ganglion of males, suggesting that CpG island methylation may be involved in inhibiting *Sp-CFSH* expression in tissue-specific and gender-variant manners. The ratio of methylated CpG island in the eyestalk ganglion of females at the early-vitellogenic stage, late-vitellogenic stage, and muscle of females at the early-vitellogenic stage, as well as the eyestalk ganglion of males at the mature stage were 8.76, 8.85, 20.5, and 16.39% (Figures 5A,C,E), respectively, and the methylation levels of the methylated CpG island was 22.22, 24.99, 45.46, and 41.67% (Figures 5B,D,F), respectively.

**Figure 6:**
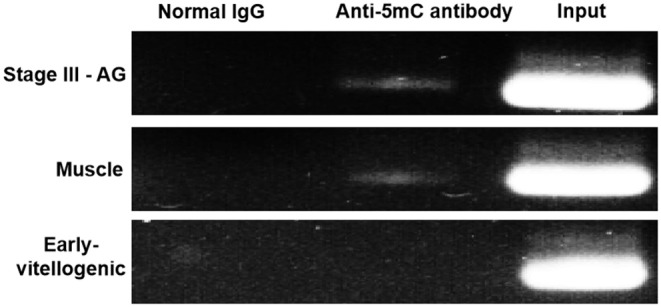
MeDIP results of CpG island methylation. Normal IgG, the immunoprecipitated DNA fragments using normal IgG; Anti-5mC antibody, the immunoprecipitated DNA fragments using anti-5mC antibody; Input, the DNA fragments. Normal IgG and Input were used as control. PCR amplified using primer sets corresponding to CpG island. Products were separated on 2.0% agarose gels and visualized by staining with GelRed. Early-vitellogenic, the eyestalk ganglion of females at early-vitellogenic stage; Stage III–AG, the eyestalk ganglion of males at stage III of AG development; Muscle, the muscle of females at early-vitellogenic stage.

MeDIP was carried out to further examine the effect of methylation on *Sp-CFSH* expression. While PCR of immunoprecipitated DNA fragments using normal IgG showed no amplified band, the immunoprecipitated DNA fragments using anti-5mC antibody were detected and showed that the amplified bands were more obvious in the muscle of females and the eyestalk ganglion of males than that in the eyestalk ganglion of females; in addition, the amplified bands of the DNA fragments were similar in three tissues (Figure 6). MeDIP further demonstrated that CpG island methylation was significantly higher in the muscle of females and the eyestalk ganglion of males than that in the eyestalk ganglion of females, suggesting that CpG island methylation may be involved in inhibiting *Sp-CFSH* expression in tissue-specific and gender-variant manners.

### Methylation of CpG Dinucleotide Sites

**Figure 7:**
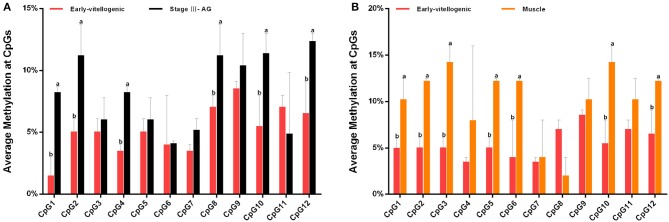
Methylation of CpG dinucleotides sites. **(A)** Comparison of the methylation of CpG dinucleotides sites in the eyestalk ganglion between females and males. **(B)** Comparison of the methylation of CpG dinucleotide sites between the eyestalk ganglion and the muscle in females. Early-vitellogenic, the eyestalk ganglion of females at early-vitellogenic stage; Stage III–AG, the eyestalk ganglion of males at stage III of AG development; Muscle, the muscle of females at early-vitellogenic stage. Different letters represent significant difference between groups (*p* < 0.05, one-way ANOVA followed by the Tukey's *post hoc* test). Each bar represents the mean ± SEM (*n* = 3).

The methylation of CpG dinucleotide sites was further studied to explore their regulation of *Sp-CFSH* expression in tissue-specific and gender-variant manners. The results showed that, compared with the eyestalk ganglion of females, there are seven CpG sites with high methylation in the muscle of females, including CpG-1, CpG-2, CpG-3, CpG-5, CpG-6, CpG-10, and CpG-12 (Figure 7A), and six CpG sites were found in the eyestalk ganglion of males with high methylation, including CpG-1, CpG-2, CpG-4, CpG-8, CpG-10, and CpG-12 (Figure 7B). Among them, CpG-1, CpG-2, CpG-10, and CpG-12 were shared between a tissue-specific manner and a gender-variant manner, suggesting that they may play an equally important role in inhibiting *Sp-CFSH* expression in two manners. Moreover, combined with the analysis of the 5′-flanking sequence of *Sp-CFSH*, CpG-1 and CpG-2 were found to be located in the binding site of the transcription factor Sp1.

### Analysis of the Binding of Sp1 With the Site-Directed Mutation (SDM)

**Figure 8:**
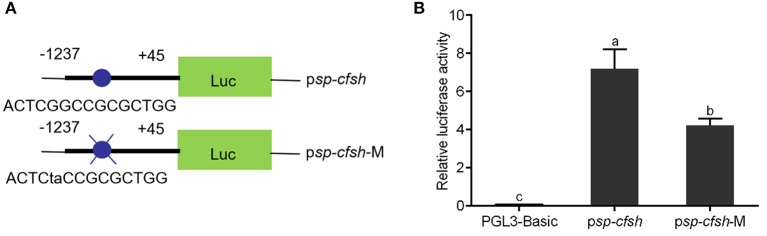
Analysis of the binding site of transcription factor Sp1 with SDM. **(A)** Schematic diagram of reporter vector; p*sp-cfsh*-M was constructed by mutating the binding site of Sp1 from p*sp-cfsh*. **(B)** The analysis of relative luciferase activity; pGL3-basic was served as the negative control. The data are presented as mean ± SEM (*n* = 3) with diverse letters indicating significant differences at *p* < 0.05.

SDM was performed to investigate whether methylation of CpG-1 and CpG-2 inhibits *Sp-CFSH* expression by blocking the binding of transcription factor Sp1. The reporter vectors were named as p*sp-cfsh* (ACTCGGCCGCGCTGG) and p*sp-cfsh*-M (ACTCTACCGCGCTGG), respectively (Figure 8A), and results showed that the promoter activity of ps*p-cfsh-*M was significantly decreased (*p* < 0.05) compared with that of ps*p-cfsh* (Figure 8B), but both of them were significantly higher than that of the negative control (pGL3-basic).

## Discussion

To date, there are few reports on the epigenetic regulatory mechanism in crustaceans. In this study, DNA methylation was shown to be involved in inhibiting *Sp-CFSH* expression in an eyestalk ganglion-specific and gender-variant pattern in the mud crab *S. paramamosain*.

To explore the regulatory mechanism of *Sp-CFSH* expression, the 5′-flanking region of *Sp-CFSH* was cloned and analyzed in this study. A total of 1,250-bp 5′-flanking regions were obtained and contained the core promoter with a TATA box, which is consistent with previous studies that there is only one transcription initiation site and a TATA box in the promoter (36, 37). Forecast analysis found some regulatory factor binding sites in the 5′-flanking regions of *Sp-CFSH*, such as GATA-1, Wt1, Sox-2, TBP, Sp1, C/EBPalp, and c-Jun. As we know, the eukaryotic promoter modulates the gene expression by the response to regulatory factor (8, 9).

In the present study, one CpG island was found in the 5′-flanking regions of *Sp-CFSH*, including 12 CpG sites, the presence of which suggests that *Sp-CFSH* expression may be regulated by DNA methylation. CpG island, as the major target for methylation, plays an important role in epigenetic regulation of gene expression (16). Most researches on DNA methylation has been done in vertebrates and plants, generally demonstrating that DNA methylation is involved in the silencing of gene expression (11–15). A study on DNA methylation in *S. paramamosain*, an important economic species of aquaculture, is completely lacking. In this study, CpG island methylation was analyzed, and the results showed that CpG island methylation was significantly higher in the muscle of females and the eyestalk ganglion of males compared to that in the eyestalk ganglion of females (Figures 5, 6), and that was inversely correlated with the *Sp-CFSH* expression (27), suggesting that methylation may be involved in inhibiting *Sp-CFSH* expression in tissue-specific and gender-variant manners by reducing the activity of the promoter (38).

The methylation of CpG dinucleotide sites including CpG-1, CpG-2, CpG-4, CpG-8, CpG-10, and CpG-12 in the eyestalk ganglion of males and CpG-1, CpG-2, CpG-3, CpG-5, CpG-6, CpG-10, and CpG-12 in the muscle of females was significantly higher than that of the eyestalk ganglion of females, suggesting that they could play a vital role in the regulation of *Sp-CFSH* expression in gender-variant and tissue-specific manners, respectively. Moreover, CpG-1, CpG-2, CpG-10, and CpG-12 have high methylation in the muscle of females and the eyestalk ganglion of males, suggesting that they may play an equally important role in the regulation of *Sp-CFSH* expression in tissue-specific and gender-variant manners. CpG-1 and CpG-2 were found to be located in the binding site of transcription factor Sp1. In addition, our results of the activity analysis of the SDM reporter vector showed that SP1, as the transcriptional activator, plays a crucial role in promoter activity. Sp1 is a vital component of the eukaryotic cellular transcriptional machinery, which fine tunes cellular functions by regulating the gene expression with GC-rich promoters. Previous studies showed that DNA methylation interferes with the binding of Sp1 to *cis* sites (39–43). In the retinoblastoma gene, methylation of the CpG island directly inhibits the binding of Sp1 (44). In extracellular superoxide dismutase (EC-SOD) gene, methylation markedly decreased Sp1-/Sp3-driven promoter activity and was, at least in part, attributable to the competition of the methyl-binding protein MeCP2 with Sp1 for the same binding sites (45). Moreover, in the Synapsin I (SYN1) gene, methylation of Sp1 *cis* sites assist RE1-silencing transcription factor (REST) in the inhibition of SYN1 transcription (46). For these reasons, it was speculated that methylation of CpG-1 and CpG-2 may be involved in inhibition of Sp1 binding or the effectiveness of repressor complex, therefore leading to a reduced *Sp-CFSH* expression in tissue-specific and gender-variant manners.

In the present study, the difference between 22.22 and 45.46%/41.67% methylation levels of CpG island (Figure 5), and the difference of 1.5–7.05% vs. 8.25–14.25% methylation levels of CpG dinucleotide sites (Figure 7) have the significant consequence in *Sp-CFSH* expression, suggesting that *Sp-CFSH* expression was sensitive to slight methylation. It has been reported that the effect of methylation on gene expression depended on several parameters, including the location of CpG dinucleotide relative to the promoter (47), the local density of methylated CpG dinucleotide (48), the strength of the promoter (48), and the dependence of promoter function on transcription factors that are sensitive to methylated CpG dinucleotide (49). In this study, methylated CpG-1 and CpG-2 were located in the binding site of transcription factor Sp1, suggesting that Sp1 may contribute to the *Sp-CFSH* expression. Previous studies have already verified that subtle changes in methylation status can induce remarkable impacts at the organismal level. For example, methylation patterns can be modulated by environmental conditions in the California mussel, *Mytilus californianus*, and the change in methylation status of LIG4 gene can be largely attributed to a single CpG site (50).

The mud crab, *S. paramamosain*, is an annual animal and reproduces once in its lifetime. The ovarian development goes through three main stages: pre-vitellogenic stage, early-vitellogenic stage, and late-vitellogenic stage (30, 31). In this study, *Sp-CFSH* expression was also found to be different in females during ovarian development. This is not the only finding that shows that *Sp-CFSH* expression is linked to ovarian development. In kuruma prawn, *Marsupenaeus japonicus*, a CFSH isoform was also found to be highly expressed in the ovary and localized to oogonia and pre-vitellogenic oocytes in vitellogenic ovaries (51). Therefore, it is speculated that *Sp-CFSH* may also be involved in the regulation of ovarian development, especially in the stimulation of oocyte vitellogenesis. To exclude the influence of environment factors on methylation, female crabs from the same sea area and season were selected in this study. Although *Sp-CFSH* expression changed remarkably during ovarian development, CpG island methylation remained stable in the eyestalk ganglion. It showed that methylation was not involved in the regulation of *Sp-CFSH* expression during ovarian development. In addition to epigenetic regulation, the synthesis and release of neurohormone can also be regulated by some neurotransmitters in crustaceans (52–56), such as serotonin (5-hydroxytryptamine, 5-HT). It has been reported that 5-HT stimulates the release of neurohormones, including the crustacean hyperglycemic hormone, red pigment-dispersing hormone, neurodepressing hormone, molt-inhibiting hormone, and red pigment-concentrating hormone in the red swamp crayfish, *Procambarus clarkii* (57–59) and the white Pacific shrimp, *Litopenaeus vannamei* (60). Recent studies also demonstrate that 5-HT can promote ovarian *MroCFSHs* expression in the giant freshwater prawn, *Macrobrachium rosenbergii* (61). Therefore, the fluctuation *Sp-CFSH* expression during ovarian development may be influenced by other endocrine regulators, such as 5-HT. In addition, post-transcriptional regulation is another important way to affect the fate of mRNA, which can be realized by various modifications of mRNA to meet the needs of different physiological states (62). The regulatory mechanism of *Sp-CFSH* expression remains to be further explored.

In summary, the 5′-flanking region of *Sp-CFSH* was first cloned and analyzed in this study. Analysis of CpG island methylation proved that DNA methylation was involved in inhibiting *Sp-CFSH* expression in eyestalk ganglion-specific and gender-variant pattern in *S. paramamosain*. Analysis of CpG dinucleotide site methylation and activity of SDM reporter vector demonstrated that methylation inhibited *Sp-CFSH* expression by blocking the binding of transcription factor Sp1. The finding suggested, for the first time, the involvement of CpG methylation in the regulation of *Sp-CFSH* expression.

## Data Availability Statement

The datasets generated for this study can be found in the National Center for Biotechnology Information, GenBank accession number: MF489232, MF489233.

## Ethics Statement

The study was approved by Xiamen University animal care committee.

## Author Contributions

QJ and HY: conceptualization. QJ and DL: methodology, investigation, and visualization. QJ: software, validation, formal analysis, data curation, and writing—original draft. QJ, HY, and HH: writing—review and editing. HY: resources, supervision and project administration. HY and GW: funding acquisition.

### Conflict of Interest

The authors declare that the research was conducted in the absence of any commercial or financial relationships that could be construed as a potential conflict of interest.

## Supplementary Material

Figure S1 Hematoxylin and eosin (HE) staining in ovaries. **(A)** pre-vitellogenic ovaries, **(B)** early-vitellogenic ovaries, **(C)** late-vitellogenic ovaries. Og, oogonia; Oc, oocyte; FC, follicle cell; n, nucleus; nu, nucleolus; yg, yolk granules.

## References

[1] Doerfler W DNA methylation-a regulatory signal in eukaryotic gene expression. J Gen Virol. (1981) 57:1–20. 10.1099/0022-1317-57-1-1 6275010

[2] Wilson C Bellen HJ Gehring WJ. Position effects on eukaryotic gene expression. Annu Rev Cell Bi. (1990) 6:679–714. 10.1146/annurev.cb.06.110190.003335 2275824

[3] Blake WJ Kærn M Cantor CR Collins JJ. Noise in eukaryotic gene expression. Nature. (2003) 422:633–7. 10.1038/nature01546 12687005

[4] Barrett LW Fletcher S Wilton SD. Regulation of eukaryotic gene expression by the untranslated gene regions and other non-coding elements. Cell Mol Life Sci. (2012) 69:3613–34. 10.1007/s00018-012-0990-9 22538991 PMC3474909

[5] Carninci P Kasukawa T Katayama S Gough J Frith MC Maeda N The transcriptional landscape of the mammalian genome. Science. (2005) 5740:1559–63. 10.1126/science.1112014 16141072

[6] Cheng J Kapranov P Drenkow J Dike S Brubaker S Patel S. Transcriptional maps of 10 human chromosomes at 5-nucleotide resolution. Science. (2005) 308:1149–54. 10.1126/science.1108625 15790807

[7] ENCODE Project consortium identification and analysis of functional elements in 1% of the human genome by the ENCODE pilot project. Nature. (2007). 447:799–816. 10.1038/nature05874 17571346 PMC2212820

[8] Smale ST Kadonaga JT. The RNA polymerase II core promoter. Annu Rev Biochem. (2003) 72:449–79. 10.1146/annurev.biochem.72.121801.161520 12651739

[9] Juven-Gershon T Hsu JY Theisen JW Kadonaga JT. The RNA polymerase II core promoter—the gateway to transcription. Curr Opin Cell Biol. (2008) 20:253–9. 10.1016/j.ceb.2008.03.003 18436437 PMC2586601

[10] Watt F Molloy PL. Cytosine methylation prevents binding to DNA of a HeLa cell transcription factor required for optimal expression of the adenovirus major late promoter. Gene Dev. (1988) 2:1136–43. 10.1101/gad.2.9.1136 3192075

[11] Ehrlich M Gama-Sosa MA Huang LH Midgett RM Kuo KC McCune A. Amount and distribution of 5-methylcytosine in human DNA from different types of tissues of cells. Nucleic Acids Res. (1982) 10:2709–21. 10.1093/nar/10.8.2709 7079182 PMC320645

[12] Herman JG Baylin SB. Gene silencing in cancer in association with promoter hypermethylation. N Engl J Med. (2003) 349:2042–54. 10.1056/NEJMra023075 14627790

[13] Shiota K. DNA methylation profiles of CpG islands for cellular differentiation and development in mammals. Cytogenet Genome Res. (2004) 105:325–34. 10.1159/000078205 15237220

[14] Suzuki MM Bird A. DNA methylation landscapes: provocative insights from epigenomics. Nat Rev Genet. (2008) 9:465–76. 10.1038/nrg2341 18463664

[15] Cedar H Bergman Y. Linking DNAmethylation and histone modification: patterns and paradigms. Nat Rev Genet. (2009) 10:295–304. 10.1038/nrg2540 19308066

[16] Rönn T Volkov P Gillberg L Kokosar M Perfilyev A Jacobsen L. Impact of age, bmi and hba1c levels on the genome-wide dna methylation and mrna expression patterns in human adipose tissue and identification of epigenetic biomarkers in blood. Hum Mol Genet. (2015) 24:3792–813. 10.1093/hmg/ddv124 25861810

[17] Bai J Gong W Wang C Gao Y Hong W Chen X. Dynamic methylation pattern of *cyp19a1a* core promoter during zebrafish ovarian folliculogenesis. Fish Physiol Biochem. (2016) 42:947–54. 10.1007/s10695-015-0187-x 26719066

[18] Feng S Cokus SJ Zhang X Chen PY Bostick M Goll G. Conservation and divergence of methylation patterning in plants and animals. Proc Natl Acad Sci USA. (2010) 107:8689–94. 10.1073/pnas.1002720107 20395551 PMC2889301

[19] Lyko F Foret S Kucharski R Wolf S Falckenhayn C Maleszka R. The honey bee epigenomes: differential methylation of brain DNA in queens and workers. PLoS Biol. (2010) 8:e1000506. 10.1371/journal.pbio.1000506 21072239 PMC2970541

[20] Bonasio R Li Q Lian J Mutti NS Jin L Zhao H. Genome-wide and caste-specific DNA methylomes of the ants *Camponotus floridanus* and *Harpegnathos saltator*. Curr Biol. (2012) 22:1755–64. 10.1016/j.cub.2012.07.042 22885060 PMC3498763

[21] Yan H Simola DF Bonasio R Liebig J Berger SL Reinberg D. Eusocial insects as emerging models for behavioural epigenetics. Nat Rev Genet. (2014) 15:677–88. 10.1038/nrg3787 25200663

[22] Yan H Bonasio R Simola DF Liebig J Berger SL Reinberg D. DNA methylation in social insects: how epigenetics can control behavior and longevity. Nucl Physiol A. (2015) 60:435–52. 10.1146/annurev-ento-010814-020803 25341091

[23] Vandegehuchte MB Kyndt T Vanholme B Haegeman A Gheysen G Janssen CR. Occurrence of DNA methylation in *Daphnia magna* and influence of multigeneration Cd exposure. Environ Int. (2009) 35:700–6. 10.1016/j.envint.2009.01.002 19249097

[24] Vandegehuchte MB Lemiere F Janssen CR. Quantitative DNA-methylation in *Daphnia magna* and effects of multigeneration Zn exposure. Comp Biochem Physiol C. (2009) 150:343–8. 10.1016/j.cbpc.2009.05.014 19486948

[25] Dovilé S Gediminas A Eimantas A Arunas L Rasa S KeStutis A Analysis of dna methylation and hydroxymethylation in the genome of crustacean *daphnia pulex*. Genes Basel. (2015) 7:1 10.3390/genes7010001 PMC4728381 26729172

[26] Zmora N Chung JS. A novel hormone is required for the development of reproductive phenotypes in adult female crabs. Endocrinology. (2014) 155:230–9. 10.1210/en.2013-1603 24280057

[27] Liu A Liu J Liu F Huang YY Wang GZ Ye HH. Crustacean female sex hormone from the mud crab *Scylla paramamosain* is highly expressed in prepubertal males and inhibits the development of androgenic gland. Front Physiol. (2018) 9:924. 10.3389/fphys.2018.00924 30065661 PMC6056722

[28] Jiang Q Lu B Lin D Huang H Chen X Ye H. Role of crustacean female sex hormone (CFSH) in sex differentiation in early juvenile mud crabs, *Scylla paramamosain*. Gen Comp Endocrinol. (2020) 289:113383. 10.1016/j.ygcen.2019.113383 31904358

[29] Liu H Cheung KC Chu KH Cell structure and seasonal changes of the androgenic gland of the mud crab Scylla paramamosain (Decapoda: Portunidae). Zool Stud. (2008) 47:720–32.

[30] Huang X Ye H Huang H Yang Y Gong J. An insulin-like androgenic gland hormone gene in the mud crab, *Scylla paramamosain*, extensively expressed and involved in the processes of growth and female reproduction. Gen Comp Endocrinol. (2014) 204:229–38. 10.1016/j.ygcen.2014.06.002 24929228

[31] Yang Y Zheng B Bao C Huang H Ye H. Vitellogenin2: spermatozoon specificity and immunoprotection in mud crabs. Reproduction. (2016) 152:235–43. 10.1530/REP-16-0188 27458256

[32] Liu YG Mitsukawa N Oosumi T Whittier RF. Efficient isolation and mapping of *Arabidopsis thaliana* T-DNA insert junctions by thermal asymmetric interlaced PCR. Plant J. (1995) 8:457–63. 10.1046/j.1365-313X.1995.08030457.x 7550382

[33] Liu YG Whittier RF. Thermal asymmetric interlaced PCR: automatable amplification and sequencing of insert end fragments from P1 and YAC clones for chromosome walking. Genomics. (1995) 25:674–81. 10.1016/0888-7543(95)80010-J 7759102

[34] Gardiner-Garden M Frommer M. CpG islands in vertebrate genomes. J Mol Biol. (1987) 196:261–82. 10.1016/0022-2836(87)90689-9 3656447

[35] Li R Zou H Jia Y Zhao R. Glucocorticoid receptor is involved in the breed dependent transcriptional regulation of mtDNA- and nuclear-encoded mitochondria genes in the liver of newborn piglets. BMC Vet Res. (2013) 9:87. 10.1186/1746-6148-9-87 23618392 PMC3644494

[36] Anish R Hossain MB Jacobson RH Takada S. Characterization of transcription from TATA-less promoters: identification of a new core promoter element XCPE2 and analysis of factor requirements. PLoS ONE. (2009) 4:e5103. 10.1371/journal.pone.0005103 19337366 PMC2659449

[37] Desfarges S Abderrahmani A Hernàndez-Novoa B Munoz M Ciuffi A. LEDGF/p75 TATA-less promoter is driven by the transcription factor Sp1. J Mol Biol. (2011) 414:177–93. 10.1016/j.jmb.2011.10.010 22019592

[38] Bewick AJ Vogel KJ Moore AJ Schmitz RJ. Evolution of dna methylation across insects. Mol Biol Evol. (2016) 34:654–65 10.1093/molbev/msw264 28025279 PMC5400375

[39] Kudo S Fukuda M. Tissue-specific transcriptional regulation of human leukosialin (CD43) gene is achieved by DNA methylation. J Biol Chem. (1995) 270:13298–302. 10.1074/jbc.270.22.13298 7768930

[40] Kudo S. Methyl-CpG-binding protein MeCP2 represses Sp1-activated transcription of the human leukosialin gene when the promoter is methylated. Mol Cell Biol. (1998) 18:5492–9. 10.1128/MCB.18.9.5492 9710633 PMC109134

[41] Kitazawa S Kitazawa R Maeda S. Transcriptional regulation of rat cyclin D1 gene by CpG methylation status in promoter region. J Biol Chem. (1999) 274:28787–93. 10.1074/jbc.274.40.28787 10497251

[42] Cao YX Jean JC Williams MC. Cytosine methylation of an Sp1 site contributes to organ-specific and cell-specific regulation of expression of the lung epithelial gene T1α. Biochem J. (2000) 350:883–90. 10.1042/bj3500883 10970805 PMC1221323

[43] Butta N Larrucea S Alonso S Rodriguez RB Arias-Salgado EG Ayuso MS. Role of transcription factor Sp1 and CpG methylation on the regulation of the human podocalyxin gene promoter. BMC Mol Biol. (2006) 7:17. 10.1186/1471-2199-7-17 16684343 PMC1481587

[44] Clark SJ Harrison J Molloy PL. Sp1 binding is inhibited by mCpmCpG methylation. Gene. (1997) 195:67–71. 10.1016/S0378-1119(97)00164-9 9300822

[45] Zelko IN Mueller MR Folz RJ. CpG methylation attenuates Sp1 and Sp3 binding to the human extracellular superoxide dismutase promoter and regulates its cell-specific expression. Free Radical Bio Med. (2010) 48:895–904. 10.1016/j.freeradbiomed.2010.01.007 20079429 PMC2838251

[46] Paonessa F Latifi S Scarongella H Cesca F Benfenati F. Specificity protein 1 (Sp1)-dependent activation of the synapsin I gene (SYN1) is modulated by RE1-silencing transcription factor (REST) and 5'-cytosine-phosphoguanine (CpG) methylation. J Biol Chem. (2013) 288:3227–39. 10.1074/jbc.M112.399782 23250796 PMC3561544

[47] Murray EJ Grosveld F Site specific demethylation in the promoter of human gamma-globin gene does not alleviate methylation mediated suppression. EMBO J. (1987) 6:2329–35. 10.1002/j.1460-2075.1987.tb02508.x 3665878 PMC553636

[48] Boyes J Bird AP. Repression of genes by DNA methylation depends on CPG density and promoter strength: Evidence for involvement of a methyl-CPG binding protein. EMBO J. (1992) 11:327–33. 10.1002/j.1460-2075.1992.tb05055.x 1310933 PMC556453

[49] Tate PH Bird AP. Effects of DNA methylation on DNA-binding proteins and gene expression. Curr Opin Genet Dev. (1993) 3:226–31. 10.1016/0959-437X(93)90027-M 8504247

[50] Chin B Characterizing the Role of DNA Methylation Patterns in the California Mussel, Mytilus californianus. Rohnert Park, CA: Deptment of Biology, Sonoma State University (2018). p. 1–38.

[51] Tsutsui N Kotaka S Ohira T Sakamoto T. Characterization of distinct ovarian isoform of crustacean female sex hormone in the kuruma prawn, *Marsupenaeus japonicus*. Comp Biochem Physiol A. (2018) 217:7–16. 10.1016/j.cbpa.2017.12.009 29277431

[52] Fingerman M Roles of neurotransmitters in regulating reproductive hormone release and gonadal maturation in decapod crustaceans. Invertebr Reprod Dev. (1997) 31:47–54. 10.1080/07924259.1997.9672562

[53] Ongvarrasopone C Roshorm Y Somyong S Pothiratana C Petchdee S Tangkhabuanbutra J. Molecular cloning and functional expression of the *Penaeus monodon* 5-HT receptor. BBA-Gene Struct Expr. (2006) 1759:328–39. 10.1016/j.bbaexp.2006.07.004 16949686

[54] Sainath SB Reddy PS Effect of selected biogenic amines on reproduction in the fresh water edible crab, *Oziotelphusa senex*. Aquaculture. (2011) 313:144–8. 10.1016/j.aquaculture.2011.01.010

[55] Swetha CH Sainath SB Reddy PR Reddy PS Reproductive endocrinology of female crustaceans: perspective and prospective. J Mar Sci. (2011) 3:1 10.4172/2155-9910.S3-001

[56] Kornthong N Chotwiwatthanakun C Chansela P Tinikul Y Cummins SF Hanna J. Characterization of red pigment concentrating hormone (RPCH) in the female mud crab (*Scylla olivacea*) and the effect of 5-HT on its expression. Gen Comp Endocrinol. (2013) 185:28–36. 10.1016/j.ygcen.2013.01.011 23376531

[57] Sarojini R Nagabhushanam R Fingerman M 5-Hydroxytryptaminergic control of testes development through the androgenic gland in the red swamp crayfish, *Procambarus clarkii*. Invertebr Reprod Dev. (1994) 26:127–32. 10.1080/07924259.1994.9672409

[58] Sarojini R Nagabhushanam R Fingerman M Mode of action of the neurotransmitter 5-hydroxytryptamine in stimulating ovarian maturation in the red swamp crayfish, *Procambarus clarkii*: an *in vivo* and *in vitro* study. J Exp Zool Part A. (1995) 271:395–400. 10.1002/jez.1402710509

[59] Yang Y Lin D Bao C Huang H Ye H. Serotonergic mechanisms of oocyte germinal vesicle breakdown in the mud crab, *Scylla paramamosain*. Front Physiol. (2019) 10:797. 10.3389/fphys.2019.00797 31275175 PMC6593242

[60] Vaca AA Alfaro J Ovarian maturation and spawning in the white shrimp, *Penaeus vannamei*, by serotonin injection. Aquaculture. (2000) 182:373–85. 10.1016/S0044-8486(99)00267-7

[61] Thongbuakaew T Suwansa-ard S Sretarugsa P Sobhon P Cummins SF Identification and characterization of a crustacean female sex hormone in the giant freshwater prawn, *Macrobrachium rosenbergii*. Aquaculture. (2019) 507:56–68. 10.1016/j.aquaculture.2019.04.002

[62] Zhao BS Roundtree IA He C. Post-transcriptional gene regulation by mRNA modifications. Nat Rev Mol Cell Biol. (2017) 18:31. 10.1038/nrm.2016.132 27808276 PMC5167638

