# Supplementary figures and images for: DNA Methylation Inhibits the Expression of CFSH in Mud Crab
Source: Front Endocrinol (Lausanne). 2020 Apr 9;11:163. doi: 10.3389/fendo.2020.00163 (PMC7160318; doi:10.3389/fendo.2020.00163)

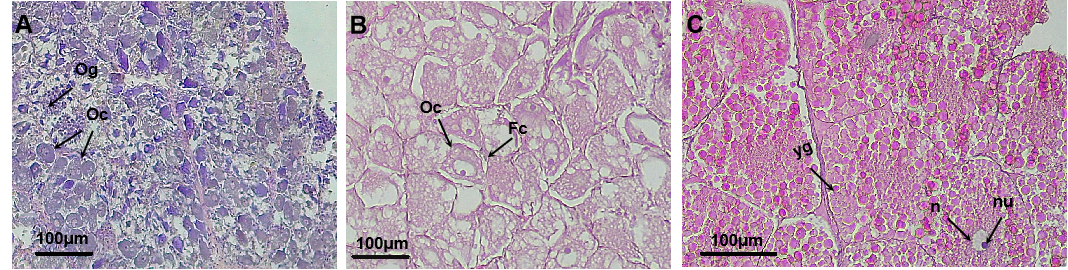

Supplement: Figure S1 — Hematoxylin and eosin (HE) staining in ovaries. (A) pre-vitellogenic ovaries, (B) early-vitellogenic ovaries, (C) late-vitellogenic ovaries. Og, oogonia; Oc, oocyte; FC, follicle cell; n, nucleus; nu, nucleolus; yg, yolk granules. [file Image_1.TIF]
